# Forage polyphenol oxidase and ruminant livestock nutrition

**DOI:** 10.3389/fpls.2014.00694

**Published:** 2014-12-08

**Authors:** Michael R. F. Lee

**Affiliations:** ^1^School of Veterinary Sciences, University of BristolBristol, UK; ^2^Rothamsted Research – North WykeOkehampton, UK

**Keywords:** polyphenol oxidase, rumen, proteolysis, lipolysis, biohydrogenation

## Abstract

Polyphenol oxidase (PPO) is predominately associated with the detrimental effect of browning fruit and vegetables, however, interest within PPO containing forage crops (crops to be fed to animals) has grown since the browning reaction was associated with reduced nitrogen (N) losses in silo and the rumen. The reduction in protein breakdown in silo of red clover (high PPO forage) increased the quality of protein, improving N-use efficiency [feed N into product N (e.g., Milk): NUE] when fed to ruminants. A further benefit of red clover silage feeding is a significant reduction in lipolysis (cleaving of glycerol-based lipid) in silo and an increase in the deposition of beneficial C18 polyunsaturated fatty acid (PUFA) in animal products, which has also been linked to PPO activity. PPOs protection of plant protein and glycerol based-PUFA in silo is related to the deactivation of plant proteases and lipases. This deactivation occurs through PPO catalyzing the conversion of diphenols to quinones which bind with cellular nucleophiles such as protein reforming a protein-bound phenol (PBP). If the protein is an enzyme (e.g., protease or lipase) the complexing denatures the enzyme. However, PPO is inactive in the anaerobic rumen and therefore any subsequent protection of plant protein and glycerol based-PUFA in the rumen must be as a result of events that occurred to the forage pre-ingestion. Reduced activity of plant proteases and lipases would have little effect on NUE and glycerol based-PUFA in the rumen due to the greater concentration of rumen microbial proteases and lipases. The mechanism for PPOs protection of plant protein in the rumen is a consequence of complexing plant protein, rather than protease deactivation *per se*. These complexed proteins reduce protein digestibility in the rumen and subsequently increase undegraded dietary protein flow to the small intestine. The mechanism for protecting glycerol-based PUFA has yet to be fully elucidated but may be associated with entrapment within PBP reducing access to microbial lipases or differences in rumen digestion kinetics of the forage and therefore not related to PPO activity.

## PPO IN FORAGES

Polyphenol oxidases (PPOs) are a group of copper metalloenzymes which include: catecholase (EC 1.10.3.2), laccase (EC 1.10.3.1), and cresolase (EC 1.14.18.1). Catecholase is the most dominant PPO in forage crops and will be referred to as PPO within this paper. Forages are typically grasses or legumes grown as feed for herbivores, usually ruminant livestock (cattle, sheep, and goats), which can be either fed directly through grazing or conserved via drying (hay) or fermentation (silage). **Table 1** summarizes screening studies for the activity of PPO protein and the presence of substrate in forage grass and legume species. Of the forage grasses and legumes screened only two, red clover (*Trifolium pratense*) and cocksfoot (*Dactylis glomerata*), have been found to have both a high PPO activity and substrate concentration. As such these two, all most exclusively, have been the focus of investigation into the impact of forage PPO on ruminant nutrition, especially the former.

**Table 1:** Summary of PPO activity and substrate concentration in the major forage species.

Species	PPO activity^1^	Substrate content^2^	Typical substrate^3^	References
**Grasses**			
Cocksfoot (*Dactylis glomerata*)	+++	+/+++	Hydroxycinnamates	Jones et al. (1995), Lee et al. (2006a), Marita et al. (2010), Parveen et al. (2010)
Hybrid ryegrass (*L. pratensis × L. multiflorum*)	++	+	Hydroxycinnamates	Lee et al. (2006a), Parveen et al. (2010)
Italian Ryegrass (*Lolium multiflorum*)	++	+	Hydroxycinnamates	Lee et al. (2006a), Parveen et al. (2010)
Maize (*Zea mays*)	++	+	Hydroxycinnamates	Harpaz and Klein (1964), Parveen et al. (2010)
Meadow fescue (*Festuca pratensis*)	+	+	Hydroxycinnamates	Jones et al. (1995), Marita et al. (2010), Parveen et al. (2010)
Perennial ryegrass (*Lolium perenne*)	++	+	Hydroxycinnamates	Jones et al. (1995), Lee et al. (2006a), Marita et al. (2010), Parveen et al. (2010)
Reed Canarygrass (*Phalaris arundinacea* L.)	+	+	Hydroxycinnamates	Jones et al. (1995), Marita et al. (2010)
Smooth bromegrass (*Bromus inermis* L.)	++	+	Hydroxycinnamates	Jones et al. (1995), Marita et al. (2010)
Tall Fescue (*Festuca arundinacea*)	+	+	Hydroxycinnamates	Jones et al. (1995), Lee et al. (2006a), Marita et al. (2010), Parveen et al. (2010)
Timothy (*Phelum pratense*)	+	+	Hydroxycinnamates	Lee et al. (2006a), Marita et al. (2010), Parveen et al. (2010)
**Legumes**			
Alfalfa (*Medicago sativa*)	–	–	–	Jones et al. (1995)
Birdsfoot trefoil (*Lotus corniculatus*)	–	+	NI	Jones et al. (1995)
Cicer milk vetch (*Astagalus cicer*)	–	+	NI	Jones et al. (1995)
Crown vetch (*Coronilla varia*)	–	+	NI	Jones et al. (1995)
Kura clover (*Trifolium ambiguum*)	–	+	NI	Jones et al. (1995)
Lespedeza (*Lespedeza cuneata)*	–	++	NI	Jones et al. (1995)
Red clover (*Trifolium pratense*)	+++	+++	Hydroxycinnamates, flavonols, flavones, isoflavones	Jones et al. (1995), Parveen et al. (2010), Lee et al. (2013)
Sainfoin (*Onobrychis viciifolia*)	–	+	NI	Jones et al. (1995)
White clover (*Trifolium repens*)	–	–/++	Flavonols, flavones, isoflavones	Jones et al. (1995), Marita et al. (2010)

*^1^PPO activities are reported as a range of units across studies [ukatal/g DM, abs/g fresh weight (FW), U/g FW] so to compare here, activities are reported relative to the species with the lowest activity in each respective study (e.g., Alfalfa): +++ = High (×100+), ++ = Medium (×10), + = Low (higher than absence), – = absence of PPO (no difference to alfalfa).*

*^2^Substrate content are reported as a range of units across the studies (abs/g fresh weight, umol/g FW) so to compare here, substrates are reported relative to the species with the lowest level in each respective study (e.g., Alfalfa): +++ = High (×2+), ++ = Medium (×1.5), + = Low (above alfalfa), – = absence of PPO (no difference to alfalfa).*

*^3^Marita et al. (2010) and Parveen et al. (2010); NI, not identified; Hydoxycinnamates in grasses tend to be chlorogenic acid whereas in red clover phaselic acid and clovamide predominate.*

**FIGURE 1:**
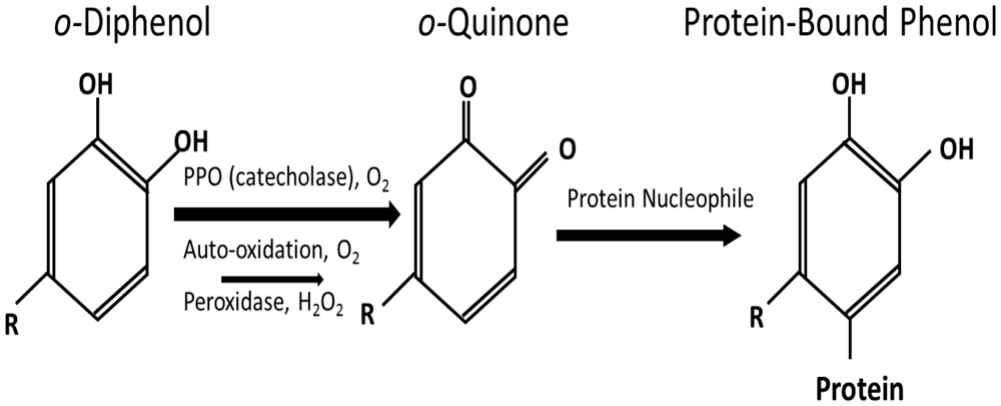
**Schematic of the polyphenol oxidase reaction to form protein-bound phenol modified from Lee et al. (2013)**.

Polyphenol oxidase catalyze the oxidation of *ortho(o)-*phenols to *o-*quinones at the expense of molecular O_2_. The reaction products, *o-*quinones, are highly reactive electrophilic molecules which act to covalently modify and cross-link a variety of cellular nucleophiles including quinone–quinone self-polymerization. *O*-quinones can also react with functional groups of proteins such as: sulfhydryl, amine, amide, indole, and imidazole substituents, forming protein-bound phenol (PBP; Bittner, 2006; **Figure 1**). The formation of *o-*quinone adducts that result in browning of various plant tissues represents the detrimental effect of PPO in post-harvest physiology and food processing and is one of the primary reasons for interest in these enzymes (Mayer, 2006). However, in forage crops such as red clover, this browning reaction has been thought to alter ruminant nutrition through improving nitrogen utilization (Albrecht and Muck, 1991; Merry et al., 2006) and the polyunsaturated fatty acid (PUFA) profile of livestock products such as milk (Lee et al., 2009a) and meat (Lee et al., 2009b).

## PPO ENZYME (ACTIVE AND LATENT)

Red clover PPO exists naturally in two forms within the chloroplast: an active form which makes up typically 5–10% of total PPO protein which shows full activity; and a latent form which makes up the vast majority of the PPO protein and requires activation. The plant therefore has two mechanisms to increase PPO activity: induction (enhancing expression of PPO genes) and activation (conversion of latent to active enzyme). Many induction factors of PPO have been confirmed across many different species, such as: pathogen invasion (Li and Steffens, 2002), attack of herbivorous insects (Steinite et al., 2004), influence of wounding (Stewart et al., 2001) and stress induced signaling compounds such as jasmonic acid (Constabel and Ryan, 1998; Gowda and Paul, 2002). Activation within the chloroplast has been shown to occur due to solubilisation, inter-conversion (Veltman et al., 1999), chemical modification such as intermolecular disulphide bridge formation, glycosylation, phenolic glucosides (Mayer and Harel, 1979), proteolytic cleavage of a pre-peptide region (Jimenez and Garcia-Carmona, 1996) and dissociation of an enzyme-inhibitor complex (Dogan et al., 2002). In grasses (e.g., cocksfoot) PPO typically is found as the active form and so methods of increasing activity are driven by increased induction as there is little latent form to activate (Winters et al., 2003). However, recently Cabiddu et al. (2014) reported the presence of a latent PPO in ryegrass in the range of 15–74% of total PPO activity, and so the same induction and activation approaches to increase active PPO may also occur in grasses.

For red clover PPO, specifically, Lee et al. (2009c) correlated degree of cell damage with activation, suggesting that loss of cellular integrity induced activation and subsequently increased PPO activity, which has agricultural importance relating to forage management (cutting and wilting during silage making). Winters et al. (2008) observed red clover PPO could be activated by its *o*-quinone substrate. Likewise Lee et al. (2013) observed that PPO enzyme when extracted from fresh leaf tissue was predominately in the latent form, however, subsequent addition of exogenous caffeic acid (*o*-diphenol) to these extracts resulted in conversion to the active form. Although the exact mechanism whereby substrate activates PPO is not known, virtually all treatments described to date that result in PPO activation (mild proteolysis, heat, pH changes, detergents, and other protein denaturing agents) would be expected to result in changes in PPO protein structure (Schmitz et al., 2008). It seems plausible that covalent attachment of an oxidized *o*-quinone and its reconfirmation into PBP could result in similar activating structural changes. In healthy red clover tissue activation is prevented by subcellular compartmentalization of PPO, present in the chloroplast and its diphenolic substrates (predominately Hydroxycinnamates, e.g., phaselic acid and clovamide; **Table 1**), which reside in the vacuoles (Mayer, 2006). This explains the correlation between degree of cell damage and PPO activation through intercellular mixing of enzyme and substrate (Lee et al., 2009c).

## PPO IN GRAZED AND CONSERVED FORAGE

For PPO to be effective in the ruminant diet a number of criteria must be satisfied. Sufficient enzyme must be present in the active form (although the exact concentration of active enzyme required is unknown), vacuolar substrate must be made available to the chloroplast localized enzyme, and O_2_ must be present (Lee et al., 2009c). These conditions are easily achieved during silage making as cellular damage during harvesting (cutting) and wilting will cause breakdown of subcellular compartmentation creating favorable conditions for activation of latent enzyme by endogenous substrates. The majority of papers reporting benefits of red clover PPO on ruminant nutrition are based on conserved (silage) feed, as red clover is predominately grown to conserve. The potential for PPO to affect ruminant nutrition under grazing has greater limitations.

**FIGURE 2:**
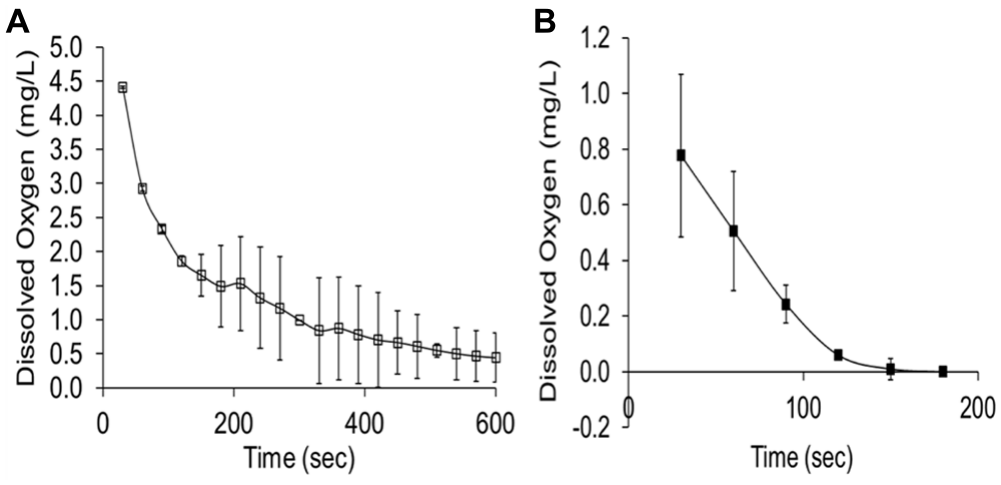
**(A)** Dissolved oxygen content of grass boluses (PRG, Perennial ryegrass) incubated in water at 39°C, *n* = 3 (± SEM). **(B)** Dissolved oxygen content of a grass (PRG) bolus incubated in rumen liquor at 39°C, *n* = 3 (± SEM; Lee et al., 2006b).

The rumen is widely considered to be anaerobic; nevertheless, rumen gas contains between 0.5 and 1.0% O_2_ by volume (McArthur and Miltimore, 1962). Czerkawski (1969) calculated that O_2_ transfer from saliva, food and diffusion from the blood of the host animal might account for 38 L of O_2_ entering the rumen of a sheep per day. Lee et al. (2009c) showed peak O_2_ levels in rumen fluid at 0.18–0.44 mg O_2_/L after swallowing returning to undetectable levels within 6–6.8 s. Within grass boluses O_2_ levels have been recorded at 5.8 mg O_2_/L declining to 0.05 mg O_2_/L after 10 min, however, in the presence of rumen fluid O_2_ levels are significantly lower and depletion of O_2_ occurs at a much quicker rate (2.5 min; **Figure 2**; Lee et al., 2006b). Although formation of the bolus alone results in O_2_ depletion, it appears to be largely due to the O_2_ scavenging nature of the microbes in the rumen fluid as confirmed by Lloyd et al. (1992) who related a low steady state of O_2_ in the rumen to the consumption of specific ruminal micro-organisms. The level to which O_2_ must fall to inhibit PPO is driven by numerous factors such as enzyme activity, substrate concentration and solubility of the O_2_. Radler and Torokfalvy (1973) reported an O_2_ requirement for grape (*Vitis vinifera*) PPO of 3.5 mg O_2_/L; whereas Gomez et al. (2006) showed inhibition of mushroom PPO at a level of 0.12 mg O_2_/L. This would be in line with the O_2_ content of boluses but below that found in the rumen liquor shortly after swallowing. It is therefore apparent that any occurrence of PPO activity during grazing of red clover would be largely confined to the period of mastication and that the amount of O_2_ brought in from the boluses would be rapidly scavenged or insufficient to maintain PPO activity.

Lee et al. (2009c) investigated activation of PPO in fresh red clover boluses and observed that the period of mastication was sufficient to activate PPO. They also observed that the degree of maturity of the red clover influenced PPO activity and PBP formation. More mature forages had higher fiber content and subsequently increased the extent of mastication, cell damage and conversion of latent to active PPO during ingestion. Therefore an increase in PBP would be expected from more mature forage, however, the more mature the crop the lower the concentration of PPO enzyme and substrate which resulted in less PBP formed, despite the higher activation from latent to active PPO of the present PPO protein.

To determine whether grazing red clover could elicit the same response on N-use efficiency (NUE) and PUFA deposition in milk as conserved red clover Lee et al. (2009a) offered dairy cows freshly cut grass, as a control, or red clover either freshly cut or wilted for 24 h, to increase opportunity for PBP formation as with making silage. They observed no difference in NUE or PUFA concentration in milk between the freshly cut and wilted red clover, suggesting that the rapid activation of PPO during mastication of the freshly cut red clover was sufficient to form adequate PBP. However, although both red clover treatments resulted in a higher PUFA content of milk, neither altered NUE compared with the grass treatment (see PPO and nitrogen metabolism for further discussion).

## PPO ACTIVITY DURING WILTING (INACTIVATION AND AUTO-OXIDATION)

Polyphenol oxidase activity during wilting has been shown to have an initial increase due to activation of the latent enzyme, which is then followed by a sharp decline (Lee et al., 2009a). This decrease in activity is a consequence of PPO inactivation predominately through *o*-quinone product binding to the PPO protein (Lee et al., 2013) which may limit the extent of PPO oxidation and ultimately PBP production. However, following self-inactivation of the PPO enzyme, by its product binding (negative feedback), oxidation of phenols continues at a lower rate. Auto-oxidation of diphenols (the non-enzymatic conversion of diphenols to quinones using molecular O_2_) has been shown to occur (Samisch, 1937; Anderson and Morris, 2001; Lim et al., 2005). In addition, peroxidases have been reported to oxidize diphenols to their corresponding quinones in the presence of hydrogen peroxide as an oxidizing agent (Vamos-Vigyazo, 1981; Miller et al., 1990; Richard-Forget and Guillard, 1997), although due to insufficient availability of hydrogen peroxide in the absence of PPO the role of peroxidase oxidation of diphenols is thought to be minor (Felton et al., 1989; Nicolas et al., 1994; Yoruk and Marshall, 2003). Recently Lee et al. (2013) demonstrated the importance of not only PPO oxidation during the initial period (0–2 h) after cutting the crop but also the continuation of oxidation by non-enzymatic/non-PPO processes in the extended wilt (2–24 h) in the formation of PBP. This highlights the importance of forage breeding strategies to increase not just PPO enzyme activity in new varieties of red clover but also substrate levels. However, the optimum level of PBP for ruminant nutrition is yet to be determined.

## PPO AND NITROGEN METABOLISM IN THE RUMEN

Polyphenol oxidase protection of plant protein in silo is related to the deactivation of plant proteases which can improve the true protein content of the silage (Broderick et al., 2001). This deactivation occurs through PPO catalyzed binding of quinones to plant protease protein where the PBP complexing denatures the enzyme. However, PPO is inactive in the anaerobic rumen and therefore any subsequent protection of plant protein in the rumen must be as a result of events that occurred pre-ingestion. Reduced activity of plant proteases in the rumen would have little impact on protein degradation due to the high concentration of rumen microbial proteases. The mechanism for PPOs protection of plant protein in the rumen is through the complexing of leaf proteins rather than protease deactivation *per se* (Winters and Minchin, 2001). Numerous studies with red clover have shown a significant reduction of rumen ammonia-N release per unit of dietary N consumed compared with other forages (Dewhurst et al., 2003; Merry et al., 2006; Vanhatalo et al., 2009). This indicates a lower degradation rate of red clover protein in the rumen due to the PPO induced complexing of protein (PBP) reducing its solubility and digestibility in the rumen. This response is associated with a greater flow of non-ammonia-N to the small intestine in essence increasing the flow of rumen undegraded dietary protein (UDP, also called by-pass protein) at the expense of rumen degradable protein (RDP). Greater flow of UDP from the rumen has the potential to increase NUE as microbial breakdown of dietary protein to ammonia in the rumen can result in significant losses across the rumen wall with subsequent formation of urea and excretion as urine. **Table 2** summarizes NUE results for dairy studies expressed as feed N conversion into milk N when feeding red clover against other forages. In all the studies where NUE was higher on red clover compared to the other forage treatment, intake of N between red clover and the other forages was comparable. In all studies where NUE on red clover was lower or no different to the other forage then N intake was significantly higher on the red clover diet. When N intake is balanced in diets, PPO protection of dietary protein can result in an increase in NUE, but surplus dietary N which is not balanced with sufficient fermentable metabolisable energy (FME), to maximize microbial protein production in the rumen, will result in a loss of N and potentially lower NUE.

**Table 2:** Comparison of red clover versus other forage treatments for nitrogen use efficiency (feed N into milk N).

Study	Fresh/silage	Comparison	Intake of N^1^	Feed N into Milk N^2^
Dewhurst et al. (2003)	Silage	G and WC	↔	↑
Broderick et al. (2000)	Silage	Alfalfa	↔	↑
Broderick et al. (2001)	Silage	Alfalfa	↔	↑
Broderick et al. (2007)	Silage	Alfalfa	↔	↑
Bertilsson and Murphy (2003)	Silage	G and WC	↑	↓
Van Dorland et al. (2006)	Silage	G and WC	↑	↓
Van Dorland et al. (2007)	Fresh	G and WC	↑	↓
Coblentz et al. (1998)	Silage	Alfalfa	↑	↔
Hoffman et al. (1997)	Silage	Alfalfa	↑	↔
Lee et al. (2009a)	Fresh	G	↑	↔

*G, grass; WC, white clover.*

*^1^Intake of N: ↔ N intake for red clover and comparison forage comparable; ↑ N intake higher for red clover than comparison forage.*

*^2^Feed N into Milk N (NUE): higher ↑, lower ↓ or no difference ↔ on red clover than the comparison forage.*

Flow of protein to the small intestine in ruminants relies on UDP, which is enhanced by PPO, and microbial protein, which relies on the balance of N release in the rumen (RDP) and supply of FME. As such microbial protein synthesis on red clover has been shown to be variable across studies based on the level of FME supplied (Dewhurst et al., 2003; Vanhatalo et al., 2009; Halmemies-Beauchet-Filleau et al., 2014). In some cases NUE on red clover diets is still lower than expected given the N supplied to the small intestine through UDP and microbial protein, supply of RDP and FME (Moorby et al., 2009; Vanhatalo et al., 2009). It has been postulated that excessive or imbalanced supply of amino acids to the mammary gland or both may limit the conversion of dietary N into milk N, with potential deficiency of the limiting amino acid methionine being implicated. It has been suggested that sulfur containing amino acids are PPO induced quinone binding points in the formation of PBP and as such may reduce their availability to the animal, i.e., overprotection resulting in reduced absorption (Lee et al., 2009a; Vanhatalo et al., 2009). Halmemies-Beauchet-Filleau et al. (2014), in a study comparing grass and red clover silage on a forage:concentrate (60:40 DM basis) diet in dairy cows, noted a progressive decline in methionine and cysteine degradability in the rumen as red clover proportion in the diet increased. Such a response is consistent with PPO overprotecting sulfur containing amino acids from metabolism in the rumen. The potential of PBP to over protect protein and the complexity of the supply of N to the duodenum of ruminants from two sources (UDP and microbial protein driven by RDP and FME availability) may explain the variability in response in NUE to red clover in the diet.

Cocksfoot PPO has been implicated with higher rumen N escape than perennial ryegrass (*Lolium perenne*) in sheep (Aufrère et al., 2003) and lower levels of rumen ammonia-N in a rumen simulation technique (RUSITEC) system compared with tall fescue (*Festuca arundinacea*; Lee et al., 2010). However, in a recent study with beef steers, cocksfoot silage showed no increase in UDP flow to the small intestine compared with perennial ryegrass silage which has significantly lower levels of PPO (Lee et al., 2014). The cocksfoot PPO enzyme and the substrate are different to that contained in red clover (Marita et al., 2010; Parveen et al., 2010). This combined with the different level and composition (amino acids) of protein, which may limit quinone binding points and ultimately extent of PBP formation, between the two forages may result in cocksfoot PPO being unable to complex protein in the same way as red clover PPO and therefore unable to elicit a protective effect in the rumen to increase UDP flow to the small intestine. Previous responses observed with cocksfoot reducing lipolysis and proteolysis *in vitro* (Lee et al., 2006a) may reflect the ability of grass PPO to deactivate plant enzymes. More research is needed to categorically confirm the inability of grass PPO to protect protein in the rumen, but current research would certainly indicate the ability of grass PPO to deactivate plant enzymes (lipases and proteases) but not to increase UDP flow from the rumen as with red clover PPO (Lee et al., 2014).

## PPO AND LIPID METABOLISM IN THE RUMEN

Two recent reviews by Van Ranst et al. (2011) and Buccioni et al. (2012) go into detail about the potential of PPO to protect glycerol-based PUFA in silo and the rumen and for more information I would direct the reader to these papers. Here I will attempt to summarize the main findings and current thinking of protection methods in the rumen. Lee et al. (2003) reported that red clover silage feeding to beef steers resulted in a greater proportion of C18 PUFA by-passing the rumen and entering the duodenum per unit of substrate supply than grass silage, i.e., lower biohydrogenation. Biohydrogenation of unsaturated fatty acids in the rumen is a product of microbial metabolism as certain bacteria require detoxification of unsaturated fatty acids and so through a process of isomerisation (*cis* to *trans* double bond conversion) and desaturation (double bond to single bond) convert unsaturated fatty acids to saturated fatty acids with a range of *trans* unsaturated intermediates (Doreau and Ferlay, 1994). Hence, the low level of PUFA and high proportion of *trans* fatty acids in ruminant products relative to animal dietary intake. Reduction of this process to improve the PUFA content of animal products has been a goal for animal scientists for over 30 years.

The action of red clover in reducing biohydrogenation was first investigated *in vitro* where it was discovered that red clover PPO reduced plant mediated lipolysis (the process of liberating PUFA as a free fatty acid from its glycerol backbone, which is a pre-requisite for biohydrogenation; Lee et al., 2004) by inhibiting plant lipases (Van Ranst et al., 2009) similar to the previously reported response with proteases (Jones et al., 1995). It was later shown that lipolysis in red clover with higher levels of PPO was also lower in the presence of microbial lipases compared with red clover which exhibited lower PPO levels (Lee et al., 2007). However, deactivation of plant lipases alone would not explain the reduction in either lipolysis or biohydrogenation in the rumen due to the high proportion of microbial lipases which would more than compensate for the loss of plant lipase activity. The lack of O_2_ in the rumen and the rapid deactivation of PPO would exclude any denaturing of microbial lipases by PPO activity in the rumen (Lee et al., 2009c, 2013). The mechanism therefore must reside in processes which occur through PPO activity pre-ingestion, as with proteins protection, or be related to other properties of red clover digestion in the rumen not linked to PPO. The true mechanism of red clovers protection of PUFA across the rumen is yet to be fully elucidated but a number of potential mechanisms have been suggested: quinone binding to the polar lipid reducing lipolysis; formation of protein complexes around glycerol-based lipid reducing access of microbial lipases; forage particle size distribution in the rumen altering digestion kinetics increasing the flow rate of red clover lipid through the rumen and so reducing microbial processing (e.g., biohydrogenation); and changes in ruminal microbial ecology resulting in lower levels of biohydrogenating bacteria on red clover based diets (Lee et al., 2003; Van Ranst et al., 2011; Halmemies-Beauchet-Filleau et al., 2013).

**Table 3** summarizes potential mechanisms associated with red clovers protection of PUFA based lipid relating to PPO either post or pre-ingestion or other mechanisms not related to PPO. Post-ingestion mechanisms have been discussed above in relation to inactivation of plant proteases and activity of PPO in the rumen which appear unlikely therefore pre-ingestion modifications appear to be the most likely or indeed mechanisms not related to PPO. Of the pre-ingestion postulated mechanisms the lease likely appears to be the potential of quinone to directly bind to glycerol-based lipid. There has been little evidence of any alteration of the membrane glycerol-based lipid through quinone binding in studies investigating the flow of lipid fractions to the small intestine in red clover fed dairy cows (Halmemies-Beauchet-Filleau et al., 2013). In addition Lee et al. (2010) reported that red clover lipid extracted from the cellular matrix showed no reduction in biohydrogenation as opposed to red clover lipid in the presence of cellular matrices when incubated in rumen fluid. This would suggest little change to the lipid itself and they postulated the protection was due to a complexing of PPO-protected protein (PBP) around glycerol-based lipid. Thereby the same mechanism that protects protein through complexing could also protect PUFA by reducing access of the membrane bound PUFA to microbial lipases within a PBP-glycerol-based PUFA matrix. This remains the only likely mechanism for PPOs protection of glycerol-based PUFA in the rumen.

**Table 3:** Postulated mechanisms and current theory on red clovers increased flow of PUFA across the rumen.

Theory	Evidence for	Evidence against	Latest thinking	Reject/unsure
**PPO Post-ingestion mechanisms**				
Deactivation of plant lipase	Lee et al. (2004), Van Ranst et al. (2009)	Harfoot and Hazlewood (1997)	Even though PPO deactivates plant enzymes it is unlikely to have an impact in the rumen due to high levels of microbial enzymes	X
Deactivation of microbial lipase	Lee et al. (2004), Van Ranst et al. (2009)	Lee et al. (2009c, 2013)	Even though PPO has been shown to deactivate a range of enzymes, in the rumen the lack of oxygen and rapid deactivation of the PPO enzyme make this mechanism improbable	X
**PPO Pre-ingestion mechanisms**				
Binding of quinone to glycerol-based lipid reducing lipolysis	Mavelli et al. (2014)	Lee et al. (2010), Halmemies-Beauchet-Filleau et al. (2013)	Although stoichiometrically binding of quinones to glycerol-based lipid is possible this has not been discovered in duodenal fluid nor has extracted glycerol-based lipid in a quinone environment shown reduced lipolysis	X
Entrapment of glycerol-based lipid with PBP reducing access to microbial lipases	Lee et al. (2010)		Protection of glycerol-based lipid within PBP formed through PPO activity may offer protection from microbial lipases through stearic hindrance of the ester bond	?
**Non-PPO mechanisms**				
Altered microbial community with altering extent of PUFA biohydrogenation	Huws et al. (2010)	Halmemies-Beauchet-Filleau et al. (2013)	Even though microbial communities differ when red clover and another forage are fed as sole feeds, when fed in combination with high starch concentrate microbial communities are similar but the greater flow in PUFA with the red clover diet is still evident	X
Altered digestion kinetics which result in lower retention times of glycerol-based lipid in the rumen	Halmemies-Beauchet-Filleau et al. (2013)		Red clover digestion kinetics indicate that glycerol-based lipid may spend less time in the rumen reducing the potential opportunity of lipolysis and biohydrogenation	?

Non-PPO potential mechanisms have suggested a role of rumen microbes or digestion kinetics. Proportions of biohydrogenation intermediates (e.g., C18:1 *trans*) have been reported to be different when either grass or red clover silages have been fed to beef steers (Lee et al., 2003, 2006c). They suggested that these differences in C18:1 biohydrogenation intermediates could relate to differences in rumen microbial communities influencing biohydrogenation pathways. Huws et al. (2010) demonstrated significantly different rumen microbial communities developed when steers were offered either grass or red clover silage. It was thought that such microbial shifts could offer an explanation for the reduction in biohydrogenation when ruminants consumed red clover, through a reduction in biohydrogenating bacterial communities. However, when red clover or grass silages are fed in combination with concentrate (60:40; forage:concentrate, DM basis) the difference in C18:1 biohydrogenation intermediates is minimal but the reduction in PUFA biohydrogenation on the red clover:concentrate diet is still observed (Halmemies-Beauchet-Filleau et al., 2013). This would suggest that the high starch concentrate would mask any forage effects which may drive microbial differences and that the reduction in biohydrogenation is not driven by the different microbial community.

Halmemies-Beauchet-Filleau et al. (2013) reported greater flow of esterified lipid at the omasum when animals consumed red clover based diets which they related to either alteration in digestion kinetics or forage particle size distribution in the rumen. The greater proportion of small particles and the subsequent increased flow rate from and reduced retention within the rumen, could explain a reduced biohydrogenation of PUFA associated with red clover. Less time for PUFA in the rumen to undergo microbial lipolysis would decrease biohydrogenation, as PUFA in the duodenum was predominately glycerol-based (esterified). However, a greater flow of glycerol-based PUFA from the rumen does not rule out the potential that PPOs binding of protein may influence the flow of the glycerol-based PUFA from the rumen. Further work is therefore required to determine the exact nature of red clover’s protection of PUFA across the rumen.

## QUESTIONS AND FUTURE WORK

In order to fully maximize the potential benefits of PPO in ruminant nutrition a more detailed understanding of how quinones complex protein is required. What drives protein complexing which protects protein in the rumen? Does the amino acid composition of the protein and the subsequent quinone binding sites influence how well the protein complexes and therefore protection in the rumen? Do certain phenolic substrates lead to a more cross-linked highly bound protein which is more protected in the rumen, e.g., mono-phenolic substrate (one binding site) versus diphenolic substrate (two binding sites)? Further studies require detailed analysis of protein complexing and amino acid binding sites to fully elucidate mechanisms as the current assay to measure PBP does not give a true account of protein complexing (Lee et al., 2013; Cabiddu et al., 2014). Although current procedures to assay PBP (Winters and Minchin, 2005) can be used to indicate PPO oxidation there are factors which result in overestimation (phenolic content of substrate and amino acids) or underestimation (loss of protein solubility) of protein complexing. Therefore future work should focus on the impact of different levels of protein complexing, measured at the protein structure level, on N and PUFA metabolism in the rumen, specifically determining the binding sites of the quinones and the different roles of contrasting phenolic substrate.

## Conflict of Interest Statement

The Guest Associate Editor, Dr. Ana Winters, declares that, despite having collaborated with author, Dr. Michael R. F. Lee, the review process was handled objectively. The author declares that the research was conducted in the absence of any commercial or financial relationships that could be construed as a potential conflict of interest.
